# The Diagnosing Challenge of a Positive ANCA Vasculitis in the Paediatric Age

**DOI:** 10.1155/2017/2962794

**Published:** 2017-12-19

**Authors:** Clara Preto, Armandina Silva, Sandra Alves, Margarida Guedes, Paula Matos, Conceição Mota, Paula Rocha, Paula C. Fernandes

**Affiliations:** ^1^Paediatric and Neonatal Intensive Care Service, Centro Materno Infantil do Norte, Centro Hospitalar do Porto, Porto, Portugal; ^2^Paediatric Service, Hospital da Senhora da Oliveira, Guimarães, Portugal; ^3^Otorhinolaryngology Service, Centro Hospitalar de Vila Nova de Gaia/Espinho, Vila Nova de Gaia, Portugal; ^4^Paediatric Service, Centro Materno Infantil do Norte, Centro Hospitalar do Porto, Porto, Portugal; ^5^Department of Paediatric Nephrology, Paediatric Service, Centro Materno Infantil do Norte, Centro Hospitalar do Porto, Porto, Portugal

## Abstract

ANCA-positive systemic vasculitides, rare in paediatric age, present multiorganic involvement. A female teenager presented with a history of subglottic stenosis diagnosed at the age of 12. From the investigation carried out, we highlight hematoproteinuria and negative ANCAs. At 15 years old, she was admitted for gastrointestinal symptoms and respiratory distress. She presented poor peripheral perfusion, pulmonary haemorrhage, respiratory failure, and severe renal insufficiency. She was started mechanical ventilation and emergency haemodialysis. The immunological study revealed ANCA MPO positive. A presumptive diagnosis of ANCA-positive vasculitis was made, and she was started corticotherapy, cyclophosphamide, and plasmapheresis. A renal biopsy, performed later, showed crescentic glomerulonephritis with chronicity signs. Positive ANCA vasculitis may progress slowly or suddenly. The diagnosis was confirmed by a biopsy; however, we can make a presumptive diagnosis based on clinical findings and in a positive ANCA test in order to start an early treatment and decrease the associated morbimortality.

## 1. Introduction

Vasculitides are disorders characterized by inflammation of the blood vessel that may occur as a primary process or secondary to an underlying disease. Apart from common vasculitides such as Henoch–Schonlein purpura and Kawasaki disease, most of the primary vasculitic syndromes are rare in childhood [1–4].

The antineutrophil cytoplasmic antibody- (ANCA-) associated vasculitides (AAV) are a group of chronic, multiorgan, and often relapsing diseases [3, 5]. The three classic AAV are granulomatosis with polyangiitis (GPA), microscopic polyangiitis (MPA), and eosinophilic granulomatosis with polyangiitis (EGPA).

EGPA is an eosinophilic-rich necrotizing vasculitis, exceedingly rare in childhood, characterized by chronic rhinosinusitis, asthma, and peripheral blood eosinophilia [4, 6]. In EGPA, ANCA is found in a minority of children [4].

GPA is characterized by a necrotizing granulomatous small and medium vessel vasculitis, and it mainly affects older children [3, 4, 7]. Patients often present constitutional symptoms (88%), renal involvement (83%), lower respiratory disease (74%), and upper airways/ear, nose, and throat involvement (70%) [7]. GPA is frequently related with antibody directed against proteinase 3 (PR3) that stains neutrophils in a cytoplasmic fashion (c-ANCA) [4, 7, 8].

MPA is a necrotizing nongranulomatous vasculitis that affects small size blood vessels [3, 4, 9]. This disease affects children significantly younger than those that GPA affects [7]. Comparatively to GPA, almost all children have constitutional symptoms (85%) in MPA; however, pulmonary manifestations are less frequent (44%) and less severe [7]. Renal involvement is also frequent (75%) and tends to be more severe than that in paediatric GPA [7]. In MPA, contrary to GPA, the upper respiratory tract is spared [4, 7]. MPA is commonly associated with elevated titers of ANCA directed at myeloperoxidase (MPO) that lead to perinuclear staining of neutrophils (p-ANCA) [4, 7, 8].

Although GPA is primarily associated with PR3-ANCA and MPA with MPO-ANCA, about 25% of the patients with GPA or MPA have the alternative ANCA [10].

The priorities in the management of a child with AAV are the prompt recognition and early treatment, as these diseases can be severe and life-threatening if not appropriately managed [11]. The diagnosis of AAV is strongly suggested by a positive ANCA test; however, 5–10% of the patients do not develop ANCA, so a negative result does not exclude a diagnosis of AAV [8, 11]. The biopsy of an affected organ remains the most definitive method to establish a diagnosis [8, 11]. Treatment comprises remission induction, with initial immunosuppressive therapy and maintenance immunosuppressive therapy for a variable period to prevent relapse [4, 5, 8, 9, 11].

Despite treatment, AAV still carries considerable disease-related morbidity and mortality mainly due to progressive renal failure or aggressive respiratory involvement [5, 6, 9].

## 2. Case Presentation

A fifteen-year-old female was transferred to the intensive paediatric care unit due to pulmonary haemorrhage, requiring mechanical ventilation, and renal insufficiency.

She has no relevant family history and has neonatal antecedents of extreme prematurity and bronchopulmonary dysplasia.

At eleven years of age, she started having episodes of respiratory distress, wheezing, coughing, and stridor that lead to multiple hospitalizations despite therapy with β2 long-acting agonists, antileukotrienes, and inhaled corticosteroids. In the exacerbations, she presented weak response to salbutamol and improvement with adrenaline and methylprednisolone. In this context, an etiological investigation was carried out at the hospital in her residential area: negative allergy screening; normal immunoglobulin and complement levels; normal alpha-1 antitrypsin level; negative sweat test; negative Ziehl–Neelsen staining and *Mycobacterium tuberculosis* culture in gastric juice; and normal upper gastrointestinal endoscopy, esophageal-gastroduodenal transit, and thoracic computed tomography.

**Figure 1:**
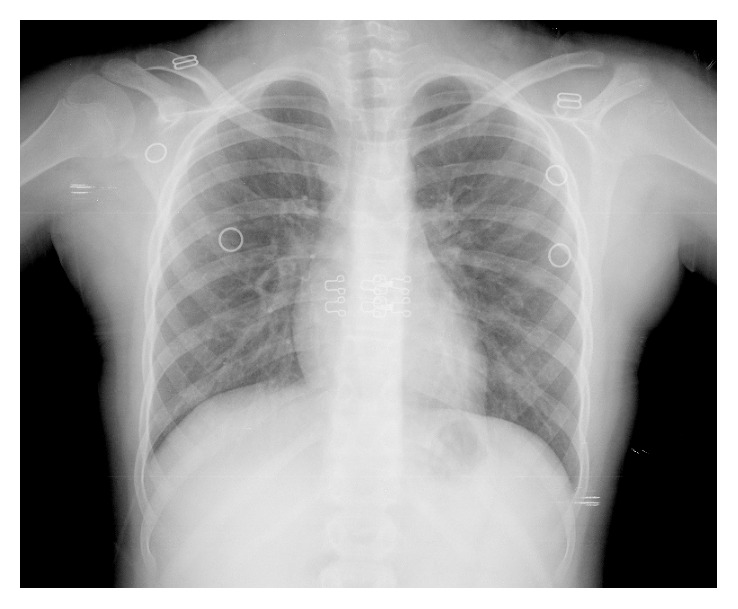
Chest radiograph showed the steeple sign.

At twelve years old, she developed progressive dysphonia showing no improvement with oral and inhaled corticosteroids. She was observed in otolaryngology consultation at a central hospital: the chest radiograph showed the steeple sign (Figure 1) and the bronchofibroscopy revealed a pleated and hardened supraglottic mucosa and subglottic stenosis. Firstly, she underwent laser surgery, and two months later, an emergency tracheotomy for respiratory insufficiency. After the tracheostomy, she needed reinterventions due to the worsening of the subglottic stenosis. From the investigation carried out in this hospital, we highlight normal angiotensin-converting enzyme level, negative antinuclear antibodies (ANA), negative ANCA (by a combination of both indirect immunofluorescence technique (IFT) and enzyme-linked immunosorbent assays (ELISAs) for PR3 and MPO), the presence of hematoproteinuria (proteins 5 mg/dl and erythrocytes > 25/field), mild changes in renal function (creatinine 0.86 mg/dl and glomerular filtration rate 65 ml/min/1.73 m^2^), and laryngeal biopsy with squamous epithelial hyperplasia and polymorphic infiltrate, without granulomas or malignancy signs.

At fourteen, the laboratory investigation was repeated at an otolaryngology consultation that disclosed the worsening of hematoproteinuria (proteins 150 mg/dl and erythrocytes > 38/µl) and renal function (creatinine 0.98 mg/dl and urea 44 mg/dl), and positivity for ANCA antibodies (IFT: ANCA 1/320, p-ANCA pattern; ELISA: anti-MPO 37 RU/ml, anti-PR3 1.4 RU/ml—normal range < 20 RU/ml).

Four months later, at the age of fifteen, she was admitted to the hospital in her residential area with a history of abdominal pain, vomiting, diarrhoea, difficulty in breathing and coughing for the past two days, and one-month history of aphonia. Examination findings revealed apyrexia, pallor, poor peripheral perfusion, and difficulty in breathing with global retraction and bilateral crepitations at pulmonary auscultation. She also presented hypertension (144/99 mmHg), generalized mild oedema, oliguria, and bleeding through the tracheostomy hole. Initial investigations revealed mixed acidosis (pH 7.01; pCO_2_ 62.3 mmHg; HCO_3_ 15.5 mmol/L; and lactate 1.1 mmol/L), severe anaemia with normal leukocyte and platelet accounts (haemoglobin 5.3 g/dl; leukocytes 12.700/uL; and platelets 318.000/uL), severe renal impairment (urea 339 mg/dl and creatinine 18 mg/dl), and hyperkalaemia (7.18 mmol/L). The remaining ionogram, liver function, coagulation screen, and C-reactive protein were within normal limits. The chest radiograph disclosed diffused opacity with ill-defined edges (like cotton wool) in the lower half of both lung fields, and ultrasound revealed moderate left pleural effusion. Bronchoscopy revealed bronchial serohematic content.

The patient's condition deteriorated rapidly, and she was transferred to the intensive paediatric care unit, in our hospital, for respiratory and renal support.

In the intensive paediatric care unit, while reviewing the patient's electronic clinical process, we detected previous analyses that had not been valued. Given the clinical picture of renal failure with hematoproteinuria associated with pulmonary haemorrhage with positive MPO-ANCA, a presumptive diagnosis of vasculitis anti-MPO ANCA positive was made, and she immediately started treatment with daily methylprednisolone (650 mg/day) and support measures (red blood cells concentrate, antihypertensives, sodium bicarbonate, calcium gluconate, salbutamol, and insulin) and haemodialysis. On the fourth day of treatment, methylprednisolone was changed to oral prednisolone (1 mg/kg/day) and she started oral cyclophosphamide (2 mg/kg/day) and plasma exchange for seven days. She also received prophylactic cotrimoxazole.

From the laboratorial investigation carried out in the intensive paediatric care unit, we highlight immunoglobulins and C4 within normal values, C3 slightly decreased (77.1 mg/dl), negative anti-glomerular basement membrane antibodies, negative ANA, and positive ANCA MPO (IFT: ANCA 1/640, p-ANCA pattern; ELISA: anti-MPO 55.8 UQ, anti-PR3 14.6 UQ—normal range < 20 UQ). Thoracic computed tomography showed areas with a bilateral ground glass appearance and hilar adenomegalies. Echocardiogram revealed a small pericardial effusion without further changes.

The patient was ventilated for a total of 13 days and remained clinically stable with progressive hypertension control and with no new episodes of pulmonary haemorrhage. She maintained oligoanuric renal insufficiency with the need of dialysis treatment. A renal biopsy was performed, and the histologic examinations revealed crescentic glomerulonephritis with a strong fibrosis degree. A pauci-immune pattern was observed in immunofluorescence microscopy.

On the thirty-third day of hospitalization, cyclophosphamide treatment was suspended due to leukopenia and induction therapy was started with rituximab (750 mg/m^2^/dose, two doses with fifteen-day breaks). She was discharged after 36 days of hospitalization with oral prednisolone that was slowly tapered.

Currently, half a year later, she maintains haemodialysis therapy; however, she has improved from the tracheal stenosis allowing her better vocal quality. There were no further complications. The ANCA became negative with reduction of the anti-MPO concentration to <2.3 UQ (normal range < 20 UQ).

## 3. Discussion

AAV are rare in the paediatric age and can progress slowly over months or explosively over days. In this case report, the long period between the onset of symptoms and the diagnosis clearly illustrates the difficulty faced in recognizing AAV.

The respiratory symptoms, present since she was eleven years old, constituted an early manifestation of the disease. Nevertheless, the diagnosis of tracheal stenosis was only made six months later, after the development of dysphonia. After this diagnosis, an etiological investigation was carried out at the otolaryngology centre and they found hematoproteinuria and mild renal insufficiency that was not properly valued since the immunological study was normal. Later, the investigation was repeated, and she presented a worsening of hematoproteinuria and renal function with positive ANCA. Nonetheless, these results were not valued before the life-threatening episode observed in the admission.

In the intensive care unit, a presumptive diagnosis of MPO-ANCA-positive vasculitis was made, allowing for the start of immediate treatment. Later, a renal biopsy was performed, and it was compatible with the previous presumptive diagnosis; however, unfortunately a high chronicity degree was also observed, and the patient was considered with end-stage renal disease.

Classification criteria of vasculitides were developed in order to standardize clinical definitions of different vasculitides but not to diagnose disease in individuals [12]. The goal was to define homogeneous populations to facilitate clinical research studies. Once childhood vasculitides are rare, experience of individual physicians in diagnosing and caring for children with chronic vasculitis is also limited, as was recognized by Ozen in an survey in which the median number of patients with an AAV diagnosed by any rheumatologist in a single year was <1 [13]. Thus, clinicians with limited experience to these particular conditions may use classification criteria to assist in the diagnosis of individual patients [13, 14]. The first classification criteria, and most widely used, were developed by the American College of Rheumatology (ACR); however, these criteria are based upon data from adult patients [15]. Once some individual criteria are more frequent or characteristic of childhood presentation (like subglottic stenoses in GPA) [16], the European League Against Rheumatism (EULAR) and the Paediatric Rheumatology European Society (PRES) proposed in 2005 a system of classification for vasculitides in children, which was validated in 2008 (EULAR/PRINTO/PRES criteria) [17, 18]. Unfortunately, due to the few cases of the less frequent forms of AAV in their population, classification criteria were not developed for MPA. European Medicines Agency (EMA) to attempt to solve these limitations developed an algorithm which includes a list of surrogate markers for GPA, reflecting those features most typical of GPA not occurring in MPA, like subglottic stenosis [19]. Thereby, this algorithm in a sequential manner applies different criteria (including ACR or EULAR/PRINTO/PRES criteria for GPA), definitions, and surrogate markers, from most specific to least specific, in a stepwise approach allowing the differentiation between CSS, GPA, and MPA [19].

Applying EULAR/PRINTO/PRES criteria, we can affirm that our patient has GPA (she fulfills four out of six GPA criteria—upper respiratory tract involvement, laryngotracheobronchial obstruction, lung involvement, ANCA positivity, and renal involvement—and only three criteria are required for the diagnosis) [17]. Although GPA is typically associated with PR3-ANCA, MPO-ANCA can be found in about 25% of these patients [10]. Thus, the presence of MPO-ANCA does not rule out GPA diagnosis as shown by the EULAR/PRINTO/PRES criteria that only included in their criteria ANCA positivity, which could be for MPO or PR3 [17].

The goal of AAV therapy is the induction of complete remission which is defined as the absence of active disease. Initial immunosuppressive therapy in AAV typically consists of glucocorticoids combined with either cyclophosphamide or rituximab [4]. In our case, and in addition to this therapy, plasmapheresis was also performed, as this child had pulmonary haemorrhage and severe renal insufficiency with the need for dialysis [4, 20, 21]. In this case report, and despite the child maintaining end-stage renal disease after induction immunosuppressive therapy, she presented tracheal and pulmonary disease remission and ANCA titers declined to become negative. There are controversies about the role of ANCA titers in determining efficacy of treatment and in the prediction of the development of a relapse. The absence of ANCA is usually evidence against a flare, but the opposite is not true [4].

There is little knowledge about the optimal treatment of AAV patients who developed severe renal insufficiency. These patients have less probability of responding to therapy compared with preserved renal function patients and additionally present an increased risk for immunotherapy adverse effects. A recent study with patients requiring dialysis at the time of diagnosis showed that, in the absence of active extrarenal vasculitis, continued immunosuppressive therapy beyond four months is very unlikely to benefit patients who remain dependent on dialysis [22]. Thus, in this case, due to the need of dialysis therapy since the admission and the strong chronicity degree observed in kidney biopsy, we decided not to administer maintenance immunosuppressive therapy.

The authors intend to point out the difficulty in diagnosing AAV and distinguishing between these diseases, as they are rare in paediatric age, their clinical features and serologies often overlap, and they have a varied clinical course, which is sometimes indolent. In this case, the diagnosis was even more difficult since the serologies were initially negative, and the child was monitored in different centres which may have hampered the clinical data integration. Although the classification criteria for AAV were not elaborated for the purpose of individual diagnosis, given the rarity and difficulty of diagnosing these diseases, they could be used by physicians for diagnosis when their experience with patients with AAV is limited [13, 14].
